# Automatic Three-dimensional Detection of Photoreceptor Ellipsoid Zone Disruption Caused by Trauma in the OCT

**DOI:** 10.1038/srep25433

**Published:** 2016-05-09

**Authors:** Weifang Zhu, Haoyu Chen, Heming Zhao, Bei Tian, Lirong Wang, Fei Shi, Dehui Xiang, Xiaohong Luo, Enting Gao, Li Zhang, Yilong Yin, Xinjian Chen

**Affiliations:** 1 School of Electronic and Information Engineering, Soochow University, Suzhou, Jiangsu, 215006, China; 2 Joint Shantou International Eye Center, Shantou University and the Chinese University of Hong Kong, Shantou, Guangdong, 515041, China; 3 Department of Ophthalmology and Visual Sciences, the Chinese University of Hong Kong, Shatin N.T., Hong Kong, 999077, China; 4 Beijing Tongren Hospital, Capital Medical University, Beijing, 100730, China; 5 School of Computer Science and Technology, Shandong University, Jinan, Shandong, 250100, China

## Abstract

Detection and assessment of the integrity of the photoreceptor ellipsoid zone (EZ) are important because it is critical for visual acuity in retina trauma and other diseases. We have proposed and validated a framework that can automatically analyse the 3D integrity of the EZ in optical coherence tomography (OCT) images. The images are first filtered and automatically segmented into 10 layers, of which EZ is located in the 7^th^ layer. For each voxel of the EZ, 57 features are extracted and a principle component analysis is performed to optimize the features. An Adaboost classifier is trained to classify each voxel of the EZ as disrupted or non-disrupted. Finally, blood vessel silhouettes and isolated points are excluded. To demonstrate its effectiveness, the proposed framework was tested on 15 eyes with retinal trauma and 15 normal eyes. For the eyes with retinal trauma, the sensitivity (SEN) was 85.69% ± 9.59%, the specificity (SPE) was 85.91% ± 5.48%, and the balanced accuracy rate (BAR) was 85.80% ± 6.16%. For the normal eyes, the SPE was 99.03% ± 0.73%, and the SEN and BAR levels were not relevant. Our framework has the potential to become a useful tool for studying retina trauma and other conditions involving EZ integrity.

Ocular trauma is a significant cause of visual impairment and blindness^1^. Commotio retinae is characterized by a grey-white discoloration or opacification of the retina after closed globe trauma, when the impact at the level of the ocular surface is transferred to the retina in the posterior segment^2^. Histopathologic studies of human and animal eyes have found that damage of the photoreceptor is a pathogenesis of commotio retinae^3^,^4^. Photoreceptors are specialized types of neurons in the retina that are capable of phototransduction. They are critical for vision because they convert light into biological signals.

Spectral-domain optical coherence tomography (SD-OCT) can produce high speed, high resolution, cross sectional 3D images and is a powerful technology for the non-invasive assessment of retinal physiology and pathology. In the SD-OCT image, the ellipsoid zone (EZ)^5^, previously called the photoreceptor inner segment/outer segment (IS/OS), is defined as the second hyper-reflective zone of the outer retina and is located just below the external limiting membrane^5^. A disruption of the EZ integrity represents damage to the photoreceptors and is generally linked with poorer vision in commotio retina^6^ and other retinal diseases^7^,^8^,^9^,^10^,^11^,^12^,^13^,^14^,^15^,^16^,^17^.

It would be very interesting to quantitatively assess photoreceptor damage by quantifying the 3D extent and the volume of EZ disruption because the EZ is a region with small thickness in the photoreceptor yet it has the potential in helping to diagnose diseases, evaluate the effect of treatment, and predict visual outcomes in patients with ocular trauma. To the best of our knowledge, this is the first work on automatic 3D detection of EZ disruption in OCT images. Some manual and/or 2D methods for 2D EZ disruption area detection have been reported^10^,^11^,^16^,^18^. Shin *et al.*^16^ manually measured the disrupted EZ length in a B scan slice. However, it was based on only a single 2D cross section image. In ref. 10 and ref. 18, the partial OCT projection image, or en face image, was developed to better visualize a map of the photoreceptor integrity and its disruption. However, the measurement of the EZ disruption area was still based on a 2D image and a manual method, which could involve subjective factors when selecting the disruption margins. Sayanagi *et al.*^11^ developed an automated EZ disruption margin detection and a weighted EZ disruption area calculation method. However, their measurement was based on the assumption that the disruption region was circular, while in fact this region may have an irregular shape. Itoh *et al.*^17^ developed an automated EZ mapping tool to assess the EZ integrity by providing en face visualization of EZ integrity, EZ-retinal pigment epithelium (EZ-RPE) height and EZ-RPE volume.

In this paper, we propose an automatic 3D framework to detect EZ disruption in macular SD-OCT scans. Machine learning classifiers have been widely used in a variety of OCT specific applications including layer segmentation^19^,^20^, drusen classification^21^, and microcystic macular edema segmentation^22^. In this paper, we apply an adaptive boosting (Adaboost)^23^,^24^,^25^ -based method to classify the pixels as disrupted or non-disrupted. The contributions of this work are as follows: (1) we propose a novel framework for a 3D, automated, and quantitative analysis of EZ integrity in retinal OCT images; and (2) because the detection of EZ disruption is a typical imbalanced classification problem^26^, we apply two strategies on two different levels to improve the classification performance. These two strategies include the Adaboost ensemble classification algorithm at the algorithm level and an under-sampling strategy at the data level.

## Results

### Data Analysis

To evaluate the performance of the proposed method, all the EZ disruption regions in the 3D SD-OCT images were manually marked by an ophthalmologist slice by slice using the ITK-SNAP software^27^ and saved as the ground truth. The leave-one-out method was used to train the Adaboost based integrated classifier models. Because the sample ratio of the majority class (non-disrupted) and the minority class (disrupted) was approximately (110 ±  256):1 on average, non-disrupted samples were randomly selected to match the disrupted ones. The EZ disruption volume was calculated by multiplying the disruption number by the voxel resolution.

The mean and 95% confidence intervals of the segmented EZ disruption region volumes were compared between eyes with retinal trauma and normal eyes. Student’s *t*-test was used to evaluate the statistical significance of the disruption volume differences between the two groups of eyes. Statistical correlation analysis and Bland-Altman plot analysis were utilized for a performance comparison between the proposed method and the ground truth.

To assess our experiments, several measures based on the segmented volume of the EZ disruption including sensitivity (SEN), specificity (SPE) and balanced accuracy rate (BAR) were adopted. These evaluation indexes are commonly used in imbalanced classification problems and are defined as below:


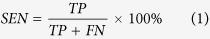



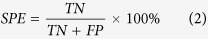



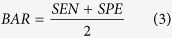


where *TP*, *FN*, *TN* and *FP* represent true positive, false negative, true negative and false negative, respectively.

### Experimental Results

**Figure 1:**
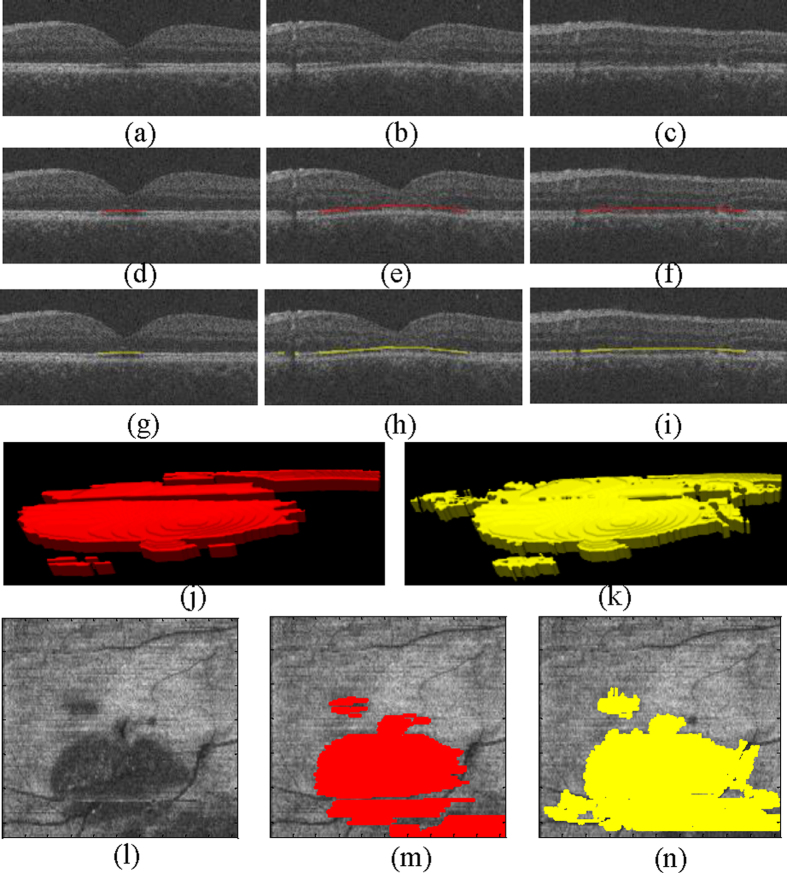
Examples of EZ disruption region detection results and ground truths for a subject with retinal trauma. The red region represents the ground truth, and the yellow region represents the segmented EZ disruption region using the proposed method. (**a–c**) Three original B-scans of an OCT volume. (**d–f**) The corresponding ground truth in the B-scans for (**a–c**), respectively. (**g–i**) The corresponding detection results using the proposed method for (**a–c**), respectively. (**j**) The ground truth in a 3D view. (**k**) The detection results in a 3D view. (**l**) The en face projection of the VOIs. (**m**) The en face projection of the ground truth (in red). (**n**) The en face projection of the detection results (in yellow).

**Figure 2:**
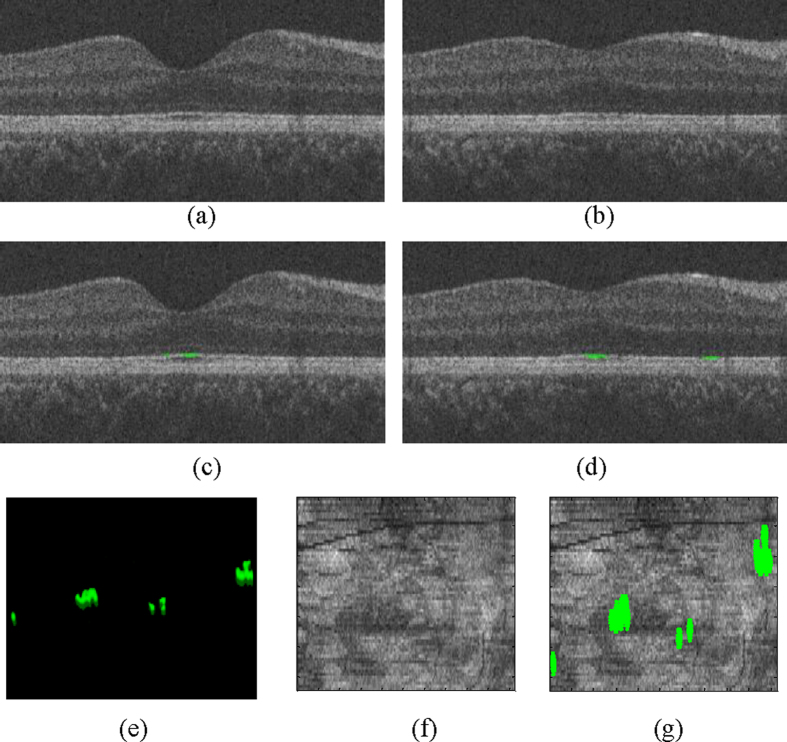
An example of the detection results using the proposed method on a normal subject. (**a,b**) The original B-scans of the OCT volume. (**c,d**) The false positive detection results (in green) using the proposed method. (**e**) All false positive detection results in a 3D view. (**f**) The en face projection of the VOIs. (**g**) The en face of the false positives (in green).

Figure 1 shows one of the detection results (Case #4 in Table 1) using the proposed framework, and the corresponding ground truth for the EZ disruption region. The en face projections of the original VOIs, ground truth, and corresponding detected EZ disruption are also shown in Fig. 1. We can see from Fig. 1 that while the proposed method detected the EZ disruption well, there were still some false positives and false negatives. The detection results for a normal eye are shown in Fig. 2. Most of the negatives were detected; however, there were still some false positives.

**Figure 3:**
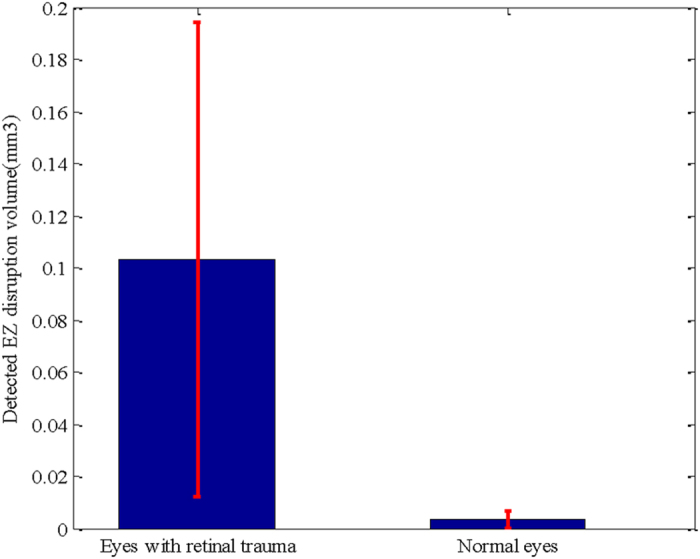
Detected EZ disruption volume comparison. The blue bars show the mean volumes, and the red error bars show the 95% confidence intervals.

The mean and 95% confidence intervals of the detected disruption volume for the normal eyes were *mean*_*normal*_ =  0.0037 *mm*^3^ and *CI*_*normal*_ =  [0.0005,0.0069]*mm*^3^, while for the eyes with retinal trauma they were *mean*_trauma_ =  0.1.35 *mm*^3^ and *CI*_*trauma*_ =  [0.0126,0.1944]*mm*^3^. The detected EZ disruption volume comparison between the normal eyes and the eyes with retinal trauma is shown in Fig. 3. Student’s *t*-test demonstrated a strong statistical significance for the detected EZ disruption volume differences between the two groups of eyes (p =  9.9112 ×  10^−8^ ≪  0.001).

Table 1 shows the detected EZ disruption volume, ground truth volume, whole EZ volume, SEN, SPE and BAR for the 15 eyes with retinal trauma. For the eyes with retinal trauma, the SEN was 85.69% ±  9.59%, the SPE was 85.91% ±  5.48%, and the BAR was 85.80% ±  6.16%. For the normal eyes, the SPE was 99.03% ±  0.73%. Because there were no true positives, the values of SEN and BAR were irrelevant.

**Figure 4:**
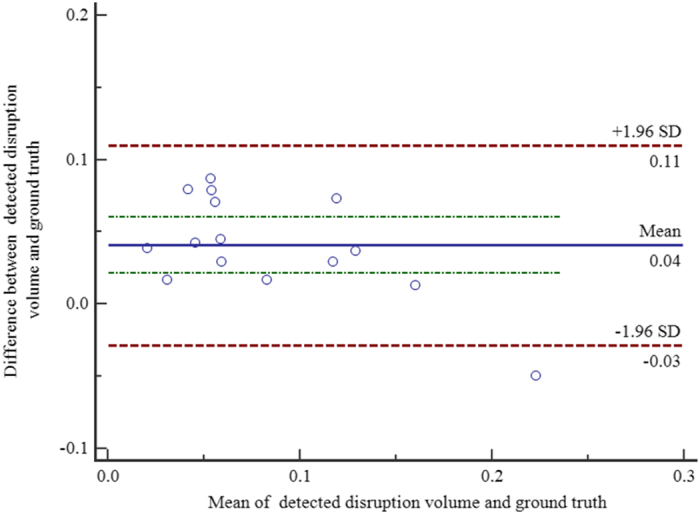
Bland-Altman plot for consistency analysis.

For the eyes with retinal trauma, the correlation between the segmented EZ disruption volume and the ground truth was *r* =  0.8795 with a significance level p <  0.0001. The 95% confidence interval for *r* was 0.6683 to 0.9595. Figure 4 shows the Bland-Altman plot for the consistency analysis between the automatic segmented EZ disruption volume and the ground truth. We can see from Fig. 4 that they are consistent.

## Discussion and Conclusion

In this study, we developed and evaluated an automatic method to detect the 3D integrity of the EZ in eyes with retinal trauma. Because the disrupted voxels in the EZ region are much less numerous than the non-disrupted ones, this leads to a typical imbalanced classification problem. To overcome this problem, an Adaboost algorithm (at the algorithm level) and dataset balance strategies (at the data level) are utilized. The vessel silhouettes and isolated points are excluded to decrease the false detections, using a vessel detector^28^ and morphological opening operations, respectively. The average detected EZ disruption volume in the eyes with retinal trauma was statistically much larger than the corresponding volume in the normal eyes (Student’s *t*-test, p =  9.9112 ×  10^−8^ <  0.001). In the eyes with retinal trauma, the SEN, SPE and BAR, using the proposed method, were 85.69% ±  9.59%, 85.91% ±  5.48%, and 85.80% ±  6.16%, respectively. There was a strong correlation between the segmented EZ disruption volume and the ground truth (*r* =  0.8795). In the normal eyes, the SPE was 99.03% ±  0.73%.

**Figure 5:**
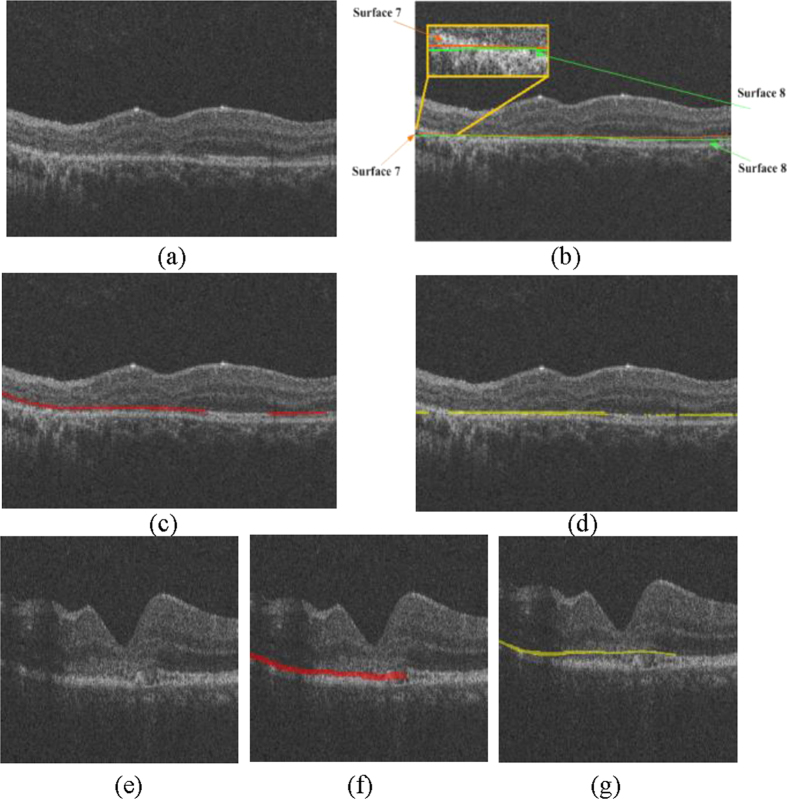
The effect of incorrect surface segmentation and poor quality in the SD-OCT image. (**a**) The original B-scans of the OCT volume. (**b**) The incorrectly segmented 7^th^ and 8^th^ surfaces. (**c**) The ground truth (in red). (**d**) The segmented EZ disruption (in yellow). (**e**) The original B-scans of the OCT volume with poor quality. (**f**) The ground truth (in red). (**g**) The segmented EZ disruption (in yellow).

This study has several limitations. (1) Although many studies have shown that the disruption extent of the EZ is an important clinical indicator for the injury degree of the photoreceptors and that EZ disruption may be closely associated with visual acuity in different eye diseases^7^,^8^,^9^,^10^,^11^,^12^,^13^,^14^,^15^,^16^,^17^, there are some controversies^29^,^30^,^31^,^32^. Whether the quantitative disruptions of the EZ have quantitative relationships with visual acuity and visual outcome has not yet been addressed. This study focuses on quantitative measurements of the EZ disruption volume; a further study will be carried out in the near future to determine the quantitative correlation, if any, of these quantitative measures to visual acuity and to the outcome of eyes with retinal trauma. (2) The sensitivity and specificity of the proposed method could also be further improved. The classification errors could be due to two reasons. i) The inaccuracy of the surface segmentation results during the image pre-processing stage may cause unreasonable extractions of VOIs. For example, Fig. 5a shows an original B-scan, and Fig. 5b shows its segmented 7^th^ and 8^th^ surfaces; both of which have lower values than the correct surfaces on the left side. Figure 5c shows the ground truth (in red), and Fig. 5d shows the detected results (in yellow). It is obvious that there are both false positives and false negatives on the left side. ii) Because of the poor quality of the SD-OCT images, the ground truths marked by the ophthalmologist are subjective, especially in the transitional region between the disrupted and the non-disrupted regions. For example, in Fig. 5e, the quality of the B-scan is very low from the left side to the central fovea area. It is difficult to decide whether the corresponding EZ is disrupted or not. Figure 5f shows the ground truth (in red), which may contain subjective preferences. Figure 5g shows the detected EZ disruption results (in yellow), which are not very consistent with the ground truth. (3) Due to the collection difficulties and poor quality of the image data, in this paper, the proposed method was tested on a sample size of 30 images dataset (15 eyes with retinal trauma and 15 normal eyes). The testing dataset is not large, however, we are still collecting more data and will validate our method on a larger dataset in the near future. (4) This work is focused on the 3D shape and volume of the EZ disruption in SD-OCT image, for more comprehensive EZ disruption analysis, the retinal information from other imaging modalities such as fundus and fundus fluorescein angiography image should be considered as well.

## Methods

### Study Subjects and Data Collection

The Institutional Review Board of the Joint Shantou International Eye Center approved this study and waived informed consent due to the retrospective nature of this study. Our study also complies with the Declaration of Helsinki. The patient records/information was made anonymous prior to analysis. The medical records and OCT database of JSIEC were searched and reviewed from February 2009 to December 2012.

In total, 15 eyes in subjects with retinal trauma and 15 eyes in normal subjects were included and underwent a macular-centred (6 ×  6 mm) SD-OCT scan (Topcon 3D OCT-1000, 512 ×  64 ×  480 voxels, 11.72 ×  93.75 ×  3.50 μ m^3^, or 512 ×  128 ×  480 voxels, 11.72 ×  46.88 ×  3.50 μ m^3^). There were 12 males and 3 females in the trauma group, with a mean age of 30.3 ±  11.3 years (range: 8–43 years). There were 9 males and 6 females in the normal group, with a mean age of 33.1 ±  10.8 years (range: 7–46 years). Subjects with other eye diseases were excluded except for those with refractive error < =  + /− 6 diopter. The raw, uncompressed data were exported from the OCT machine in “.fds” format for analysis.

### Overview of the EZ Disruption Detection Method

The proposed method consists of three main parts: pre-processing, classification, and post-processing. In the pre-processing step, the SD-OCT images are first denoised using fast bilateral filtering^33^. Then, the SD-OCT volume is automatically segmented into 10 intra-retinal layers using the multi-scale 3D graph-search approach^34^,^35^,^36^,^37^,^38^,^39^,^40^, which produces 11 surfaces. The retina in the original SD-OCT volume is flattened, and the 11^th^ surface (the bottom of the retinal pigment epithelium) is used as the reference plane. The EZ region between the 7^th^ and 8^th^ surfaces is extracted, which is the volume of interest (VOI) for our analysis. In the classification step, five categories, from a total of 57 features, are extracted for each voxel in the VOIs. Then, principle component analysis (PCA) is adopted for feature selection. Because the disrupted voxels (the minority) in the VOIs are far less numerous than the non-disrupted ones (the majority), it is a typical imbalanced classification problem. To improve the performance of the classification, we apply the following two strategies in the classification training: (1) an Adaboost algorithm is adopted to train some weak classifiers into an integrated strong classifier at the algorithm level; and (2) the majority samples are randomly under-sampled at the data level. In the classifier testing step, every voxel in the VOIs is classified as disrupted or not disrupted. In the post-processing step, the blood vessel silhouettes are identified and excluded by a vessel detector^28^ and the isolated points are excluded by morphological operations to avoid false detections. Finally, the volume of the disrupted EZ is calculated.

### Denoising by Bilateral Filtering

Speckle noise is the main noise in OCT images^41^, and it affects the performance of image processing and classification. In this paper, we propose applying the bilateral filtering^42^ method for denoising because it can remove speckle noise from images effectively while maintaining edge-like features. We have used a fast approximation algorithm^33^ to reduce the computation time without significantly impacting the bilateral filtering result. Each B-scan (X–Z image) of the OCT images is smoothed separately by bilateral filtering.

### Segmentation of the Intra-retinal Layer and the EZ Region

**Figure 6:**
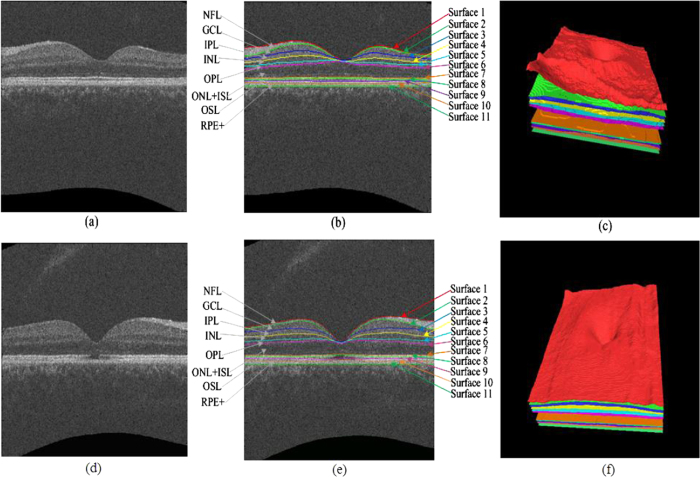
Segmentation results of 11 intra-retinal surfaces (10 layers) on a normal eye and an eye with retinal trauma. (**a**) B-scan of a normal eye. (**b**) Segmentation results of the normal eye, nerve fibre layer (NFL), ganglion cell layer (GCL), inner plexiform layer (IPL), inner nuclear layer (INL), outer plexiform layer (OPL), outer unclear layer (ONL) + inner segment layer (ISL), outer segment layer (OSL), and retinal pigment epithelium complex (RPE+ ). (**c**) Three-dimensional rendering of the segmented surfaces for the normal eye. (**d**) B-scan of an eye with retinal trauma. (**e**) Segmentation results of the eye with retinal trauma. (**f**) Three-dimensional rendering of the segmented surfaces for the eye with retinal trauma.

The filtered SD-OCT volume is then automatically segmented into 10 intra-retinal layers using the multi-scale 3D graph-search approach^34^,^35^,^36^,^37^,^38^,^39^,^40^, which produces 11 surfaces (see Fig. 6). Then, all the surfaces are smoothed using thin plate splines. The retina in the original SD-OCT volume is flattened by adjusting the A-scans up and down in the z-direction, where the 11^th^ surface (the bottom of the retinal pigment epithelium) is used as a reference plane because of its robustness. Then, the EZ regions between the 7^th^ and 8^th^ surfaces are extracted as the volumes of interest (VOIs).

### Feature Extraction

For classification, the following five types of low-level features are extracted: normalized intensity, block mean, block standard deviation, the absolute intensity difference in the 13 directions to be described later (step =  1, 2), and the grey-level co-occurrence matrix (GLCM) based features (contrast, correlation, energy and homogeneity in 13 directions). Therefore, 57 features are extracted, which are listed in Table 2.

The normalized intensity represents the voxel’s grey level. As shown in Fig. 6, the intensity level of the disrupted region of the EZ is lower than the intensity level of the non-disrupted region. Therefore, if a voxel’s normalized intensity level is low, it has a higher probability of being classified as a disruption, and vice versa.

The block mean and block standard deviation represent the average intensity level and the variance of the intensity level, respectively, in the local region centred around the voxel (region of 5 ×  5 ×  5 voxels). The absolute intensity difference in 13 directions represents the variance of the intensity between the centre voxel and its neighbours in 13 directions. Let *α*_1_ stand for the angle between the X-axis and the projection direction on the X-Y plane, and let *α*_2_ stand for the angle between the X-Y projection direction and the Z-axis. The 13 directions can be described as follows: (*α*_1_, *α*_*2*_) =  (0, 90°), (45°, 90°), (90°, 90°), (135°, 90°), (0, 45°), (180°, 45°), (90°, 45°), (−90°, 45°), (0, 0), (45°, 45°), (135°, 45°), and (−135°, 45°). The block mean, block standard deviation and absolute difference in the 13 directions can be used to distinguish the boundary between the disrupted and non-disrupted regions.

The grey-level co-occurrence matrices (GLCMs) for the 3D volumetric data describe the spatial dependence of grey levels across multiple slices^43^,^44^. The 3D method searches for other grey levels in the 13 directions (mentioned above) across multiple planes and constructs 13 GLCMs. Here, the GLCMs in the 13 directions of every 5 ×  5 ×  5 block are constructed. Then, the following four features are calculated: (1) the contrast, which measures the local contrast of the volumetric image and is expected to be higher when a large grey-level difference occurs more frequently; (2) the correlation, which provides a correlation between the two voxels in a voxel pair and is expected to be higher when the grey levels of a voxel pair are more correlated; (3) the energy, which measures the number of repeated voxel pairs and is expected to be higher if the occurrence of repeated voxel pairs is higher; and (4) the homogeneity, which measures the local homogeneity of a voxel pair and will be larger when the grey levels of each voxel pair are more similar.

### Feature Selection

Based on above definitions, we have a total of 57 features extracted for each voxel in the VOIs. To reduce the dimensionality of the feature vector and describe the inter-correlated quantitative dependence of the features, a feature selection procedure based on the PCA is performed. In our experiments, the first 10 principle components are selected as the new features; they represent more than 90% of the information in the original features.

### Adaboost Algorithm and the Under-sampling Based Integrated Classifier

In this study, the number of non-disrupted samples in the EZ region is far greater than the number of disrupted ones. The disrupted EZ samples and non-disrupted EZ samples belong to the minority and majority classes, respectively. This is a typical imbalanced classification problem, which means the class distribution is highly skewed. Most traditional single classifiers, such as the support vector machine, the k-nearest neighbour classifier, quadratic discriminate analysis, and the decision tree classifier, tend to show a strong bias towards the majority class and do not work well for this type of problem because they aim to maximize the overall accuracy. The Adaboost algorithm based integrated classifier^23^,^24^,^25^ is one solution to overcome this problem at the algorithm level; it integrates multiple weak classifiers into a strong classifier and is therefore more sensitive to the minority. Hence, the Adaboost algorithm is adopted in this study.

To further improve the classification performance at the data level, the training datasets are balanced by under-sampling majority samples. In the training step, the Adaboost algorithm-based classifier model is calculated according to leave-one-out cross-validation, using all the disrupted samples and an equivalent number of randomly selected non-disrupted samples. In the testing stage, each voxel in the VOIs is classified as disrupted or non-disrupted using the trained Adaboost model.

### Post-processing

**Figure 7:**
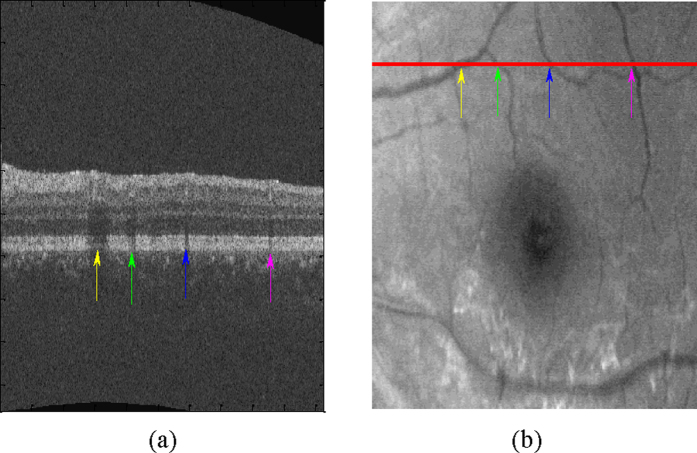
Vessel silhouettes appear as similar low-intensity regions in the EZ disruption regions. (**a**) The arrows indicate vessel silhouettes in one of the slices of the retinal OCT image. (**b**) The red line indicates the location of the slice shown in (**a**) at en face project image of the retina. The locations where vessels cross the slice and the locations of the vessel silhouettes in the same slice correspond one-to-one using differently coloured arrow pairs.

The vessel silhouettes in the EZ have lower values of intensity (see Fig. 7), and the voxels in these regions may be falsely classified as disrupted. The vessel silhouettes are identified and detected based on a vessel detector^28^. As in the outer retina (EZ to RPE), the vessel silhouettes offer excellent contrast; only those voxels between the EZ and RPE are selected and each pixel in the 2D projection image is the average in the z-axis direction of the selected voxels at that particular x, y location in the OCT volume. Then, the vessel silhouettes are segmented using a KNN classifier. If the detected EZ disruption regions have the same x and y location as the vessel silhouettes, these regions are regarded as normal and removed as false detections. Due to the physiological connectivity of the EZ disrupted/non-disrupted regions, isolated disrupted/non-disrupted voxels are eliminated through morphological opening operations, where the shape of the structural element is set as ball with a radius of 5 voxels.

## Additional Information

**How to cite this article**: Zhu, W. *et al.* Automatic Three-dimensional Detection of Photoreceptor Ellipsoid Zone Disruption Caused by Trauma in the OCT. *Sci. Rep.* **6**, 25433; doi: 10.1038/srep25433 (2016).

## Figures and Tables

**Table 1:** The detected EZ disruption volume, ground truth volume, whole EZ volume, SEN, SPE and BAR for 15 eyes with retinal trauma.

Eyes withretinal trauma	Disruption volume (mm^3^)		EZ volume (mm^3^)	SEN (%)	SPE (%)	BAR (%)
	Detection result	Ground truth				
1	0.0666	0.0238	0.4352	94.81	86.73	90.77
2	0.0394	0.0223	0.4264	81.46	79.11	80.28
3	0.1474	0.1104	0.4534	90.17	86.04	88.10
4	0.1315	0.1020	0.4846	95.92	91.32	93.62
5	0.1980	0.2472	0.4616	71.99	90.64	81.32
6	0.1663	0.1532	0.4277	92.00	90.79	91.39
7	0.0400	0.0010	0.4584	89.96	88.78	89.37
8	0.0933	0.0139	0.4310	65.12	79.80	72.46
9	0.0912	0.0202	0.4807	93.02	84.28	88.65
10	0.0809	0.0358	0.4621	70.04	86.91	78.47
11	0.0811	0.0016	0.4562	88.76	74.32	81.54
12	0.1554	0.0819	0.4972	91.54	86.17	88.86
13	0.0913	0.0742	0.4812	90.69	93.82	92.26
14	0.0737	0.0438	0.4632	88.39	89.95	89.17
15	0.0970	0.0096	0.4553	81.47	79.99	80.73
**Mean ± std**	**0.1035 ± 0.0464**	**0.0627 ± 0.0683**	**0.4583 ± 0.0216**	**85.69 ± 9.59**	**85.91 ± 5.48**	**85.80 ± 6.16**

**Table 2:** The features extracted in the feature extraction stage.

Featurenumber	Feature name	Details
1	Normalized intensity	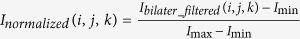 (*i, j, k*) represents the (*i, j, k*) th voxel in the VOIs.
2	Block mean	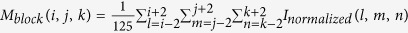
3	Block standard deviation	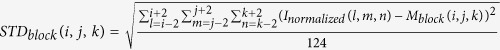
4, 5	Absolute intensity difference in 13 directions	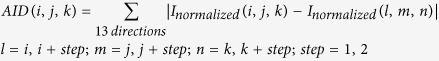
6–18	GLCM based contrast (in 13 directions)	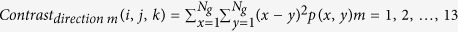 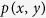 represents the (*x*, *y*) th entry in the GLCM, 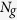 represents the number of distinct grey levels in the quantized image. Here, 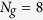 .
19–31	GLCM based correlation (in 13 directions)	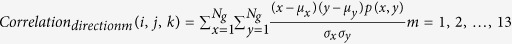 *μ*_*x*_, *μ*_*y*_, *σ*_*x*_ and *σ*_*y*_ are the means and standard deviations of the *x* row and the *y* column, respectively.
32–44	GLCM based energy (in 13 directions)	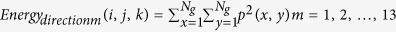
45–57	GLCM based homogeneity (in 13 directions)	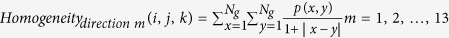

## References

[b1] Kuhn F., Mester V., Berta A. & Morris R. Epidemiology of severe eye injuries. United States Injury Registry (USEIR) and Hungarian Eye Injury Registry (HEIR). Ophthalmologe 95, 332–343 (1998). 9643026 10.1007/s003470050282

[b2] Berlin R. Zur sogenannten commotio retinae. Klin. Monatsbl. Augenh. 11, 42–78 (1873).

[b3] Sipperley J. O., Quigley H. A. & Gass D. M. Traumatic retinopathy in primates: the explanation of commotio retinae. Arch. Ophthalmol. 96, 2267–2273 (1978). 718521 10.1001/archopht.1978.03910060563021

[b4] Mansour A. M., Green W. R. & Hogge C. Histopathology of commotio retinae. Retina 12, 24–28(1992). 1565867 10.1097/00006982-199212010-00006

[b5] Staurenghi G., Sadda S., Chakravarthy U. & Spaid R. F. Proposed lexicon for anatomic landmarks in normal posterior segment spectral-domain optical coherence tomography: the IN• OCT consensus. Ophthalmology 121, 1572–1578 (2014). 24755005 10.1016/j.ophtha.2014.02.023

[b6] Chen H. *et al.* Prediction of visual prognosis with spectral-domain optical coherence tomography in outer retinal atrophy secondary to closed globe trauma. Retina 33, 1258–1262 (2013). 23508077 10.1097/IAE.0b013e31827b63ba

[b7] Saxena S., Srivastav K., Cheung C. M., Ng J. Y. W. & Lai T. Y. Y. Photoreceptor inner segment ellipsoid band integrity on spectral domain optical coherence tomography. Clin. Ophthalmol. 8, 2507–2522 (2014). 25525329 10.2147/OPTH.S72132 PMC4266419

[b8] Hangai M. *et al.* Three-dimension imaging of macular holes with high-speed optical coherence tomography. Ophthalmology 114, 763–773 (2007). 17187861 10.1016/j.ophtha.2006.07.055

[b9] Inoue M. *et al.* Spectral-domain optical coherence tomography images of inner/outer segment junctions and macular hole surgery outcomes. Graefes Arch. Clin. Exp. Ophthalmol. 247, 325–330 (2009). 19018552 10.1007/s00417-008-0999-9

[b10] Jaeryung O., William E. S., Harry W. F. J., Giovanni G. & Brandon L. Photoreceptor inner/outer segment defect imaging by spectral domain OCT and visual prognosis after macular hole surgery. Invest. Ophth. Vis. Sci. 51, 1651–1658 (2010). 10.1167/iovs.09-4420 19850825

[b11] Sayanagi K., Ikuno Y., Soga K. & Tano Y. Photoreceptor inner and outer segment defects in Myopic Foveoschisis. Am. J. Ophthalmol. 145, 902–908 (2008). 18342829 10.1016/j.ajo.2008.01.011

[b12] Spaide R. F., Koizumi H. & Freund K. B. Photoreceptor outer segment abnormalities as a cause of blind spot enlargement in acute zonal occult outer retinopathy-complex diseases. Am. J. Ophthalmol. 146, 111–120 (2008). 18439564 10.1016/j.ajo.2008.02.027

[b13] Baba T. *et al.* Correlation of visual recovery and presence of photoreceptor inner/outer segment junction in optical coherence images after successful macular hole repair. Retina 28, 453–458 (2008). 18327138 10.1097/IAE.0b013e3181571398

[b14] Kitaya N., Hikichi T., Kagokawa H., Takamiya A. & Yoshida A. Irregularity of photoreceptor layer after successful maculor hole surgery prevents visual acuity improvement. Am. J. Ophthalmol. 138, 308–310 (2004). 15289151 10.1016/j.ajo.2004.03.004

[b15] Gomes N. L. *et al.* comparison of fundus autofluorescence and retinal structure in patients with stargardt disease. Invest. Ophth. Vis. Sci. 50, 3953–3959 (2009). 10.1167/iovs.08-2657 PMC2749553 19324865

[b16] Shin H. J., Lee S. H., Chung H. & Kim H. C. Association between photoreceptor integrity and visual outcome in diabetic macular edema. Graefes Arch. Clin. Exp. Ophthalmol. 250, 61–70 (2012). 21874345 10.1007/s00417-011-1774-x

[b17] Itoh Y., Vasanji A. & Ehlers J. P. Volumetric ellipsoid zone mapping for enhanced visualisation of outer retinal integrity with optical coherence tomography. *Br. J. Ophthalmol.* Online First, published on July 22, 2015, DOI: 10.1136/bjophthalmol-2015-307105 (2015). 10.1136/bjophthalmol-2015-307105 PMC4936524 26201354

[b18] Kiernan D. F. *et al.* En face spectral-domain optical coherence tomography outer retinal analysis and relation to visual acuity. Retina 32, 1077–1086 (2012). 22466459 10.1097/IAE.0b013e31823c23bc PMC3707390

[b19] Lang A. *et al.* Retinal layer segmentation of macular OCT images using boundary classification. Biomed. Opt. Express 4, 1133–1152 (2013). 23847738 10.1364/BOE.4.001133 PMC3704094

[b20] Antony B. J. *et al.* A combined machine-learning and graph-based framework for the segmentation of retinal surfaces in SD-OCT volumes. Biomed. Opt. Express 4, 2712–2728 (2013). 24409375 10.1364/BOE.4.002712 PMC3862166

[b21] Dufour P., Zanet S. D., Wolf-Schnurrbusch Y. & Kowal J. Classification of drusen positions in optical coherence tomography data from patients with age-related macular degeneration. Conf. Proc. 21st Intl. Conf. Pattern. Recog. 2012, 2067–2070 (2012).

[b22] Lang A. *et al.* Automatic segmentation of microcystic macular edema in OCT. Biomed. Opt. Express 6, 155–169 (2015). 25657884 10.1364/BOE.6.000155 PMC4317118

[b23] Freund Y. & Schapire R. E. Experiments with a new boosting algorithm. Conf. Pro. 13th Intl. Conf. Mach. Learn. 1996, 148–156 (1996).

[b24] Freund Y. & Schapire R. E. Game theory, on-line prediction and boosting. Conf. Proc. 9th Annu. Conf. Comput. Learn. Theory 1996, 325–332 (1996).

[b25] Schapire R. E. & Singer Y. Improved boosting algorithms using confidence-rated predictions. Mach. Learn. 37, 297–336 (1999).

[b26] He H. B. & Garcia E. A. Learning from imbalanced data. IEEE Trans. Knowl. Data Eng. 21, 1263–1284 (2009).

[b27] Yushkevich P. A. *et al.* User-guided 3D active contour segmentation of anatomical structures: Significantly improved efficiency and reliability. NeuroImage 31, 1116–1128 (2006). 16545965 10.1016/j.neuroimage.2006.01.015

[b28] Niemeijer M., Garvin M. K., Ginneken B., Sonka M. & Abràmoff M. D. Vessel segmentation in 3D spectral OCT scans of the retina. Conf Proc SPIE Med. Imaging 2008, 69141R (2008).

[b29] Villate N., Lee J. E., Venkatraman A. & Smiddy W. E. Photoreceptor layer features in eyes with closed macular holes: optical coherence tomography findings and correlation with visual outcomes. Am. J. Ophthalmol. 139, 280–289 (2005). 15733989 10.1016/j.ajo.2004.09.029

[b30] Suh M. H., Seo J. M., Park K. H. & Yu H. G. Associations between macular findings by optical coherence tomography and visual outcomes after epiretinal membrane removal. Am. J. Ophthalmol. 47, 473–480 (2009). 19054492 10.1016/j.ajo.2008.09.020

[b31] Chang L. K., Koizumi H. & Spaid R. F. Disruption of the photoreceptor inner segment-out segment junction in eyes with macular holes. Retina 28, 969–975 (2008). 18698299 10.1097/IAE.0b013e3181744165

[b32] Moshfeghi A. A., Flynn H. W. Jr, Elner S. G., Puliafito C. A. & Gass J. D. M. Persistent outer retinal defect after successful macular hole repair. Am. J. Ophthalmol 139, 183–184 (2005). 15652846 10.1016/j.ajo.2004.06.082

[b33] Paris S. & Durand F. A fast approximation of the bilateral filter using a signal processing approach Int. J. Comput. Vision 81, 24–52 (2009).

[b34] Li K., Wu X., Chen D. Z. & Sonka M. Optimal surface segmentation in volumetric images-a graph-theoretic approach. IEEE Trans. Pattern Anal. Mach. Intell. 28, 119–134 (2006). 16402624 10.1109/TPAMI.2006.19 PMC2646122

[b35] Garvin M. K. *et al.* Intraretinal layer segmentation of macular optical coherence tomography images using optimal 3-D graph search. IEEE Trans. Med. Imaging 27, 1495–1505 (2008). 18815101 10.1109/TMI.2008.923966 PMC2614384

[b36] Lee K. *et al.* Segmentation of the optic disc in 3-D OCT scans of the optic nerve head IEEE Trans. Med. Imaging 29, 159–168 (2010). 19758857 10.1109/TMI.2009.2031324 PMC2911797

[b37] Chen X. J. *et al.* Three-dimensional segmentation of fluid-associated abnormalities in retinal OCT: probability constrained graph-search-graph-cut. IEEE Trans. Med. Imaging 31, 1521–1531 (2012). 22453610 10.1109/TMI.2012.2191302 PMC3659794

[b38] Chen X. J. *et al.* Quantification of external limiting membrane disruption caused by diabetic macular edema from SD-OCT. Invest. Ophth. Vis. Sci. 53, 8042–8048 (2012). 10.1167/iovs.12-10083 PMC3517271 23111607

[b39] Chen X. J. *et al.* Quantitative analysis of retinal layer optical intensities on three-dimensional optical coherence tomography. Invest. Ophth. Vis. Sci. 54, 6846–6851 (2013). 10.1167/iovs.13-12062 PMC5963175 24045992

[b40] Chen H. Y. *et al.* M. Quantitative analysis of retinal layers’ optical intensities on 3D optical coherence tomography for central retinal artery occlusion. Sci. Rep. 5, 9269 (2015). 25784298 10.1038/srep09269 PMC4363859

[b41] Zhang X. M., Li L., Zhu F., Hou W. G. & Chen X. J. Spiking cortical model-based nonlocal means method for speckle reduction in optical coherence tomography images. J. Biomed. Opt. 19, 066005 (2014). 24919448 10.1117/1.JBO.19.6.066005

[b42] Tomasi C. & Manduchi R. Bilateral filtering for gray and color images. Conf. Proc. IEEE Intl. Conf. Comput. Vis. 1998, 839–846 (1998).

[b43] Xu Y., Sonka M., McLennan G., Guo J. & Hoffman E. A. MDCT-based 3-D textural classification of emphyema and early smoing related lung pathologies. IEEE Trans. Med. Imaging 25, 464–475 (2006). 16608061 10.1109/TMI.2006.870889

[b44] Haralick R. M., Shanmugam K. & Dinstein I. Textural features for image classification. IEEE Trans. Syst. Man Cybern. SMC- 3, 610–621 (1973).

